# Biomechanical Impact of Phosphate Wasting on Articular Cartilage Using the Murine Hyp Model of X‐linked hypophosphatemia

**DOI:** 10.1002/jbm4.10796

**Published:** 2023-07-11

**Authors:** Carolyn M Macica, Steven M Tommasini

**Affiliations:** ^1^ Connecticut Children's Research Institute Hartford CT USA; ^2^ Department of Pharmacology Yale University School of Medicine New Haven CT USA; ^3^ Department of Orthopaedics and Rehabilitation Yale University School of Medicine New Haven CT USA

**Keywords:** BIOMECHANICS, CELL/TISSUE SIGNALING – ENDOCRINE PATHWAYS, CHONDROCYTE AND CARTILAGE BIOLOGY, DISEASES AND DISORDERS OF/RELATED TO BONE, DISORDERS OF CALCIUM/PHOSPHATE METABOLISM, ARTICULAR CARTILAGE, ORTHOPAEDICS, OSTEOARTHRITIS, PTH/Vit D/FGF23

## Abstract

Degenerative osteoarthritis (OA) is recognized as an early‐onset comorbidity of X‐linked hypophosphatemia (XLH), contributing to pain and stiffness and limiting range of motion and activities of daily living. Here, we extend prior findings describing biochemical and cellular changes of articular cartilage (AC) in the phosphate‐wasting environment of XLH to determine the impact of these changes on the biomechanical properties of AC in compression and potential role in the etiology of OA. We hypothesize that despite increased proteoglycan biosynthesis, disruption of the mineralized zone of AC impacts the mechanical properties of cartilage that function to accommodate loads and that therapeutic restoration of this zone will improve the mechanical properties of AC. Data were compared between three groups: wild type (WT), Hyp, and Hyp mice treated with calcitriol and oral phosphate. EPIC microCT confirmed AC mineral deficits and responsiveness to therapy. MicroCT of the Hyp subchondral bone plate revealed that treatment improved trabecular bone volume (BV/TV) but remained significantly lower than WT mice in other trabecular microstructures (*p* < 0.05). Microindentation AC studies revealed that, compared with WT mice, the mean stiffness of tibial AC was significantly lower in untreated Hyp mice (2.65 ± 0.95 versus 0.87 ± 0.33 N/mm, *p* < 0.001) and improved with therapy (2.15 + 0.38 N/mm) to within WT values. Stress relaxation of AC under compressive loading displayed similar biphasic relaxation time constants (Tau_fast_ and Tau_slow_) between controls and Hyp mice, although Tau_slow_ trended toward slowed relaxation times. In addition, Tau_fast_ and Tau_slow_ times correlated with peak load in WT mice (*r* = 0.80; *r* = 0.78, respectively), whereas correlation coefficient values for Hyp mice (*r* = 0.46; *r* = 0.21) improved with treatment (*r* = 0.71; *r* = 0.56). These data provide rationale for therapies that both preserve AC stiffness and recovery from compression. The Hyp mouse also provides unique insight into determinants of structural stiffness and the viscoelastic properties of AC in the progression of OA. © 2023 The Authors. *JBMR Plus* published by Wiley Periodicals LLC on behalf of American Society for Bone and Mineral Research.

## Introduction

Degenerative osteoarthritis/osteoarthrosis (OA) affects millions of the aging adult population in the United States^(^ ^1^ ^)^ and is recognized as a comorbidity of adult X‐linked hypophosphatemia (XLH).^(^ ^2^, ^3^, ^4^, ^5^, ^6^, ^7^, ^8^, ^9^ ^)^ Prior studies have shown that adult XLH is characterized by progressive musculoskeletal comorbidities including early‐onset, pervasive degenerative OA with marginal osteophytes and subchondral sclerosis.^(^ ^5^, ^6^, ^10^ ^)^ OA significantly impacts activities of daily living, physical function, and results in a pain‐limited range of motion (ROM) in this population.^(^ ^3^, ^5^, ^6^, ^11^ ^)^

Over a lifetime, a relatively thin layer of synovial joint articular cartilage (AC) tissue provides a smooth, lubricated surface for low‐friction articulation of long bones. Articular cartilage also permits deformation and absorbs imposed forces, while accommodating the distribution and transmission of loads to the underlying subchondral bone. The extracellular matrix (ECM) and hierarchical zonal arrangement define the biomechanical properties of articular cartilage as a two‐phase viscoelastic material.^(^ ^12^ ^)^ Positioned at the end of long bones, the function of articular cartilage is influenced by the structural hierarchy of the tissue such that the stiffness, ranging from the unmineralized zone to mineralized zone to subchondral bone, is arranged by increasing structural stiffness. This arrangement has been proposed to permit the mineralized zone of cartilage to function as a transitional zone to reduce stress at the interface between cartilage and subchondral bone.^(^ ^13^, ^14^, ^15^ ^)^

In addition to the graded continuum of increasing material stiffness, cartilage is also a biphasic viscoelastic tissue, meaning that both the solid and fluid matrix contribute to the mechanical properties of the tissue and ability to adapt to loads.^(^ ^16^ ^)^ Solid matrix deformation of large proteoglycans, restrained by stiff fibrillar collagen, contribute to the accommodation of compressive and elastic forces. The solid matrix additionally provides frictional resistance to the movement of interstitial water in response to increases in the hydrostatic pressures of loading, dissipating energy within the cartilage compartment. Studies conducted in Hyp mice, a murine model of XLH OA, revealed that there is a fundamental disruption in the normal architecture of articular cartilage, characterized by loss of the mineralized zone of articular cartilage and an upregulation of extracellular matrix proteoglycans (PGs) with sulfated glycosaminoglycan side chains.^(^ ^4^ ^)^

The early phases of OA in the general population have been attributed to alterations in the normal distribution of load on the cartilages, followed by alterations in the biochemical properties of cartilage,^(^ ^17^ ^)^ resulting in decreased compressive stiffness and increased permeability, and, ultimately, irreversible cartilage degeneration that impacts joint structure and function. Likewise, the development of early‐onset OA in patients with XLH has largely been attributed to the abnormal alignment and distribution of load on weight‐bearing joints. For example, both varus and valgus deformities impact the load on the medial or lateral tibiofemoral compartment, respectively. Notwithstanding, the contribution of the hypophosphatemic environment and accompanying changes to the structural architecture of the cartilages in the etiology of OA has been suggested^(^ ^4^ ^)^ but is currently unknown.

Here, we investigate the impact of the phosphate‐wasting environment and loss of the mineralized zone of articular cartilage on the biomechanical properties of articular cartilage (AC) in the etiology of adult OA. In addition, we consider whether the upregulation of PG biosynthesis previously reported in Hyp mouse AC is sufficient to maintain the mechanical properties of cartilage in response to compressive loading.

## Materials and Methods

### Study design and analysis

Hyp mice of the C57BL/6 strain (and age‐matched C57BL/6 controls) were obtained in‐house in the Yale University School of Medicine Animal Care Facility and experimental protocols approved by Yale IACUC. All animals were maintained on normal rat chow and in accordance with the NIH's Guide for the Care and Use of Laboratory. Additionally, the Yale University Institutional Animal Care and Use Committee approved all animal procedures. The breeding strategy employed was HYP Female × wild‐type (WT) males such that offspring resulted in a mix of WT and Hyp offspring, allowing experiments to be conducted with littermate Hyp and WT controls for comparison. A mix of both sexes (approximately 2/3 male:1/3 female) were used to conduct experiments, as the XLH phenotypic findings are similar, as reported in humans.^(^ ^18^ ^)^ The left knee of littermate Hyp and WT mice were used for microindentation or EPIC microCT studies. Contralateral right knees were used for histological staining. For microCT analysis of subchondral bone, the left knee of Hyp, Hyp‐treated, and WT mice litters were used. Sample size estimates were determined by previously reported data that Hyp mice had a significant thinning of articular cartilage compared with WT controls as quantified by EPIC‐CT (control: 0.096 ± 0.015 mm; mean ± standard deviation; HYP: 0.051 ± 0.006 mm), a standardized difference of 7.5 SD.^(^ ^4^ ^)^ To detect such a standardized difference (80% power, 5% significance) between genetic background only 3 mice/group were required, based on *n* = 2(2.8 S/Δ)^2^, where S = standard deviation and Δ = difference in means. To account for potential increase variability in the unloading groups, a minimum of 4 animals per group were used to detect differences in cartilage thickness. Nonparametric statistics were used to provide for more robust analyses given the relatively small sample sizes. To preserve statistical power, the number of comparisons between groups was limited. The primary analysis that directly tested differences between them compared WT and Hyp controls to each other via a nonparametric one‐way ANOVA (Kruskal–Wallis); slopes of linear regression load versus time constant (Tau) lines were compared using global regression, equivalent to ANCOVA analysis (GraphPad Prism 9.0, GraphPad, La Jolla, CA, USA). Percent differences between groups were expressed as means and maximum/minimum values and individual data points plotted as box plots. EPIC microCT data (total cartilage thickness, ratio of partitioned unmineralized volume/total volume, and ratio of partitioned mineralized volume/total volume were analyzed using an unpaired *t* test. Data presented in tables were expressed as mean ± SD.

#### Equilibrium partitioning of an ionic contrast agent via μCT (EPIC‐CT)

EPIC‐CT was performed to confirm the mineralization status of the joint using a Scanco 35 microCT instrument (Scanco Medical, Brüttisellen, Switzerland), as described previously,^(^ ^4^ ^)^ but using IOHEXOL (5‐(N‐(2,3‐dihydroxypropyl)acetamido)‐2,4,6‐triiodo‐N,N′‐bis(2,3‐dihydroxypropyl)isophthalamide, Sigma‐Aldrich, St. Louis, MO, USA) as the contrast agent to partition cartilages. After harvest, the tibia was immersed in an IOHEXOL PBS solution of 350 mg/mL for 30 minutes and briefly rinsed. EPIC‐microCT was performed at the Yale Core Center for Musculoskeletal Disorders (YCCMD) microCT facility using a microCT 35 (Scanco Medical) and imaged in air at 6‐micron isometric voxel size with the X‐ray tube set at a peak electric potential of 45 kVp.

### Histochemical safranin‐O staining

A subsample of ipsilateral tibias were demineralized and prepared for safranin‐O proteoglycan staining, as previously described.^(^ ^4^ ^)^ Tissue was fixed in 4% buffered paraformaldehyde for 48 hours at 4°C, decalcified in daily changes of 7% EDTA/PBS solution at pH 7.1 for 14 days at 4°C, and washed with PBS. Tissues were paraffin embedded and sectioned to a thickness of 5 μm. Histochemical staining of sulfated proteoglycans was assessed on rehydrated deparaffinized sections using safranin‐O/fast green staining. Briefly, sections were stained with Weigert's iron hematoxylin working solution (1% hematoxylin/7.25% acidified ferric chloride) for 5 minutes, washed, and followed by 0.001% fast green solution for 10 minutes, then quickly rinsed with 1% acetic acid solution. Slides were then stained in 0.1% safranin‐O solution for 7 minutes, then dehydrated and mounted with resinous medium. All histological images were captured using Olympus (Center Valley, PA, USA) Stream image analysis software on a microscope fitted with a DP22 2.8‐megapixel digital camera.

#### Mechanical testing/microindentation

Topographical stiffness mapping and average stiffness values of the proximal tibia were calculated by microindentation on freshly harvested tibia using a Mach 1‐1 Model Mechanical Tester. Settings for experiments were as follows: Z‐contact velocity, mm/s: 0.1; contact criteria, gf: 0.0375; stage limit, mm: 10; scanning grid, mm: 0.075; indentation amplitude, mm: 0.03; indentation velocity, mm/s: 0.03; relaxation time, s: 20–60 s; gap distance from surface, mm: 0; load limit, gf: 100. Average tibial cartilage thickness was approximately 100–150 μm such that an indentation amplitude of 30 μm results in a 20%–30% strain.

This foundation of these studies is based on findings first reported by the authors.^(^ ^4^ ^)^ Previous studies from our lab have shown a significant restoration in the zones of articular cartilage by treatment with calcitriol and oral phosphate. To study the impact of treatment on the biomechanical properties of articular cartilage, 3‐week‐old Hyp mice were treated with 0.1 μg/kg calcitriol, every other day for 9 weeks and water supplemented with phosphate salts (1.93 g/L).

At sacrifice, tibial tissues were harvested and wrapped in gauze soaked with PBS at 4°C. Microindentation mapping and analysis was performed as described by Gardner‐Morse and colleagues.^(^ ^19^ ^)^ Tissue‐relaxation curves were analyzed with a nonlinear fit of a curve with a biphasic decay to obtain time constants (GraphPad Prism 9.0), a phase of fast decay (Tau_fast_), and a phase of slow decay (Tau_slow_). For comparison of peak load versus relaxation time constants, the peak load (the force required to reach a maximum indentation amplitude 0.03 mm, *y*‐axis) of every microindentation point for WT, Hyp, and Hyp‐treated mice across the tibial plateau was plotted as a function of Tau_fast_ or Tau_slow_ (*x*‐axis), respectively.

## Results

### Articular cartilage structure

**Fig. 1:**
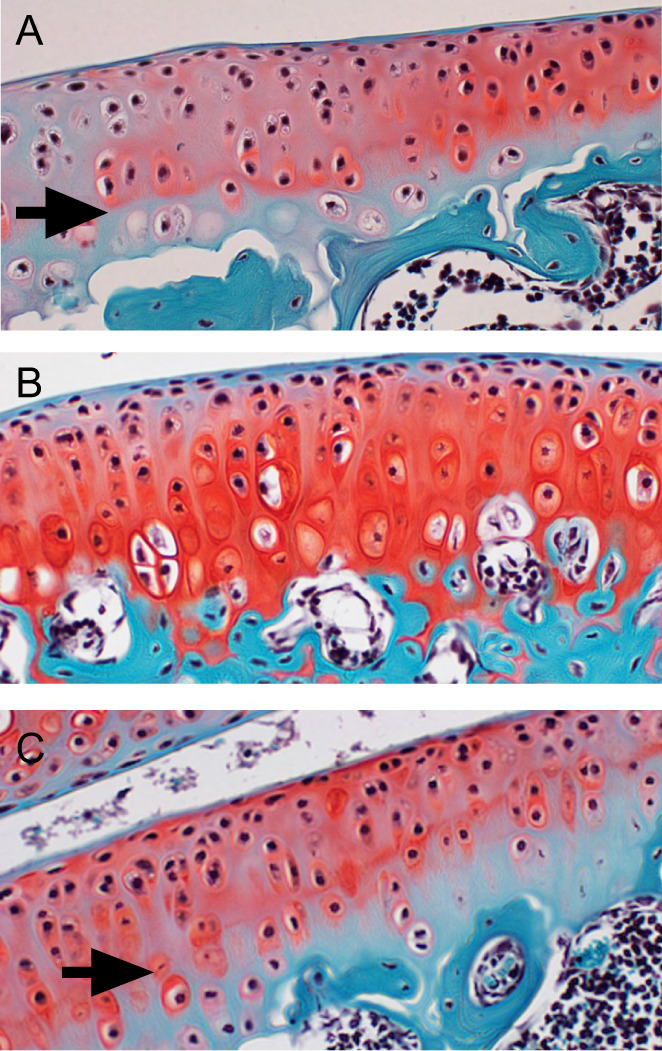
Histochemical staining of sulfated proteoglycans by safranin‐O. (*A*) Articular cartilage staining of control mice reveals distinctive proteoglycan (PG) distribution, demarcated by the tidemark boundary between unmineralized and mineralized cartilage (arrow). (*B*) In contrast, cartilage of Hyp mice was heavily stained and PGs diffusely distributed throughout the articular surface to the subchondral bone plate, with no evident tidemark. (*C*) Both PG staining intensity and tidemark (arrow) are restored in Hyp mice treated with oral phosphate and calcitriol.

EPIC‐microCT partitioning of articular cartilage studies were conducted to confirm both disruption of the tibial articular cartilage mineralized zone in the phosphate‐wasting environment and restoration of the mineralized zone with treatment in advance of biomechanical studies.^(^ ^4^ ^)^ EPIC microCT revealed that total cartilage volume was significantly higher in 12‐week‐old Hyp mice relative to WT controls (Table 1, *p* = 0.03) as previously described.^(^ ^4^ ^)^ After treatment with calcitriol and oral Pi for 9 weeks post‐weaning, volume was restored to within WT values and significantly different from untreated Hyp mice (Table 1, *p* = 0.008). In addition, there was a significant decrease in the ratio of mineralized volume to total volume (MV/TV) in Hyp mice relative to WT controls (Table 1, *p* = 0.002). After treatment, articular cartilage MV/TV in Hyp mice was significantly improved relative to untreated Hyp mice (Table 1, *p* = 0.004), consistent with previously reported data.^(^ ^4^ ^)^ In addition, both UV/TV (unmineralized to total volume ratio of cartilage) and the total cartilage thickness of treated Hyp mice were restored to within the range of WT controls (Table 1, *p* > 0.05). In addition, as reported,^(^ ^4^ ^)^ these data are supported by safranin‐O histochemical staining, a quantitative and specific stain for glycosaminoglycans.^(^ ^20^ ^)^ PGs were upregulated throughout the entire articular cartilage volume and associated with a loss in the tidemark, the boundary between the unmineralized chondrocytes and hypertrophic chondrocytes of the mineralized zone.^(^ ^21^, ^22^ ^)^ Consistent with EPIC microCT data, treatment resulted in normalization of both PG levels and histological tidemark (Fig. 1).

**Table 1:** EPIC MicroCT Partitioning of Cartilage in 12‐Week‐Old Mice

Tibial cartilage	Total CartTh (mm)				UV/TV (mm^3^/mm^3^)				MV/TV (mm^3^/mm^3^)			
	Mean	SD	*n*	*p*	Mean	SD	*n*	*p*	Mean	SD	*n*	*p*
WT control	0.027	0.012	2		0.214	0.089	2		0.418	0.014	2	
Hyp untreated	0.045	0.007	5	0.0321^a^	0.415	0.029	5	0.0032^a^	0.150	0.069	5	0.002^a^
Hyp treated	0.030	0.001	5	0.0008^b^	0.298	0.013	5	0.0001^b^	0.265	0.028	5	0.003^b^

Abbreviations: CartTh = cartilage thickness; MV/TV = mineralized volume to total volume; UV/TV = unmineralized volume to total volume; WT = wild type.

^a^ WT control versus Hyp‐untreated.

^b^ Hyp‐untreated versus Hyp‐treated.

We next conducted trabecular morphometry analysis of subchondral tibial bone in Hyp mice to determine the impact of treatment on subchondral bone as a potential determinant of structural stiffness. Unlike the beneficial effect of treatment on AC mineral in Hyp mice, microCT analysis of subchondral bone showed a relatively modest impact of treatment on the microarchitecture of subchondral bone compared with untreated Hyp mice. Significant improvement in subchondral bone of treated Hyp mice were found in the fraction of bone volume (BV/TV; *p* = 0.05) and bone mineral density (BMD; *p* = 0.02) relative to untreated Hyp mice, whereas all other parameters were not significantly different (Table 2). In addition, only BMD (*p* = 0.13) and bone surface area relative to total bone volume (BS/BV; *p* = 0.07) were restored to within WT values. Values for BV/TV, trabecular connectivity, trabecular number (Tb.N), trabecular spacing (Tb.Sp), and tissue mineral density (TMD) in Hyp mice remained significantly different (*p* < 0.05) from WT controls (Table 2) despite treatment. MicroCT data of subchondral bone thus permitted the unique opportunity to determine the impact of improved cartilage mineralization in the setting of persistent subchondral plate osteomalacia on the biomechanical properties of cartilage.

**Table 2:** MicroCT of the Tibial Subchondral Bone Plate in Response to Treatment, Mean (SD)

Sample	BV/TV (%)	Tb.N (1/mm)	Tb.Th (mm)	Tb.Sp (mm)	BMD (gm/cm^3^)	TMD (gm/cm^3^)	BS/BV (%)
Hyp (*n* = 5)	0.10 (0.05)	3.21 (0.79)	0.05 (0.007)	0.35 (0.12)	815 (28)	214 (55)	56 (11.9)
Hyp‐treated (*n* = 5)	0.18 (0.05)^a^	3.73 (0.35)	0.05 (0.007)	0.29 (0.03)	864 (26)^a^	272 (23)	50 (9.6)
WT (*n* = 2)	0.35 (003)^b^	5.90 (0.97)^b^	0.07 (0.009)	0.17 (0.03)^b^	912 (48)	410 (46)^b^	32 (6.0)

Abbreviations: BMD = bone mineral density; BS/BV = bone surface area relative to total bone volume; BV/TV = trabecular bone volume; Tb.N = trabecular number; Tb.Sp = trabecular spacing; Tb.Th = trabecular thickness; TMD = tissue mineral density; WT = wild type.

^a^ *p* <0.05 Hyp‐untreated versus Hyp‐treated.

^b^ *p* <0.05 Hyp‐treated versus WT.

### Articular cartilage stiffness

**Fig. 2:**
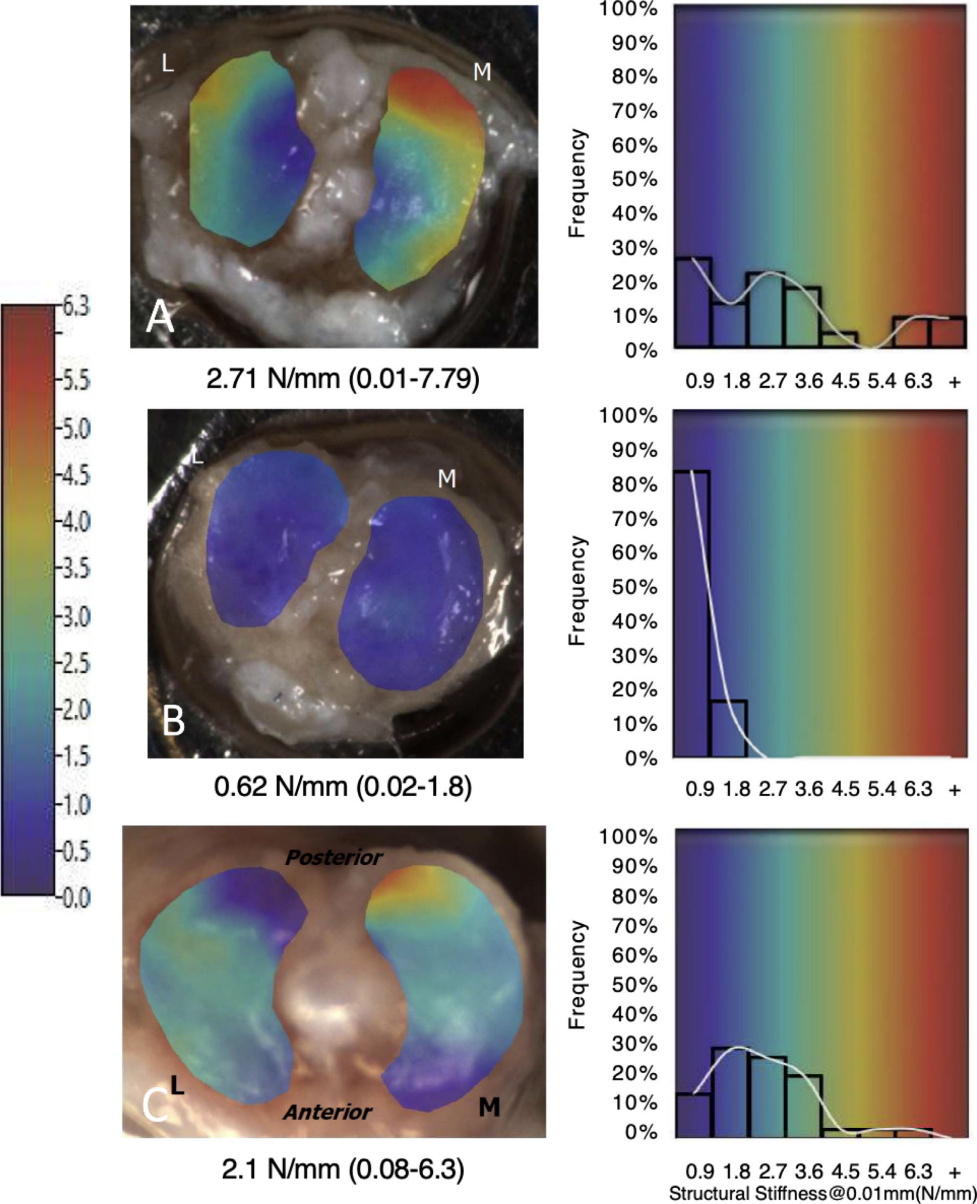
Representative mapping and stiffness of medial (M) and lateral (L) tibial plateau of Hyp and wild‐type (WT) mice as assessed by microindentation. Compared with WT (*A*), the mean structural stiffness (N/mm) of tibial anticular cartilage (AC) is significantly compromised in Hyp mice (*B*) and is responsive to calcitriol/Pi therapy (*C*). Total data are summarized in Table 3.

**Fig. 3:**
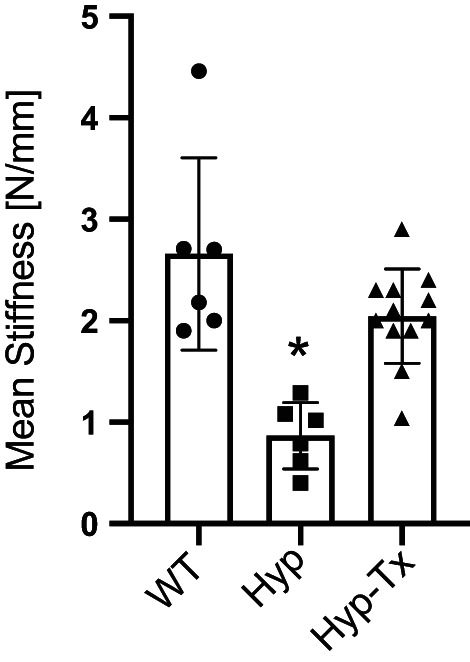
Mean structural stiffness of tibial articular cartilage. Compared with WT, the mean structural stiffness (N/mm) is significantly compromised in Hyp mice (*p* = 0.0014) but is responsive to calcitriol/Pi therapy (Hyp versus Hyp‐Tx, *p* < 0.0001).

The stiffness of tissue in response to a mechanical stress is a characteristic that defines the functional properties of AC, a tissue that has evolved to accommodate weight‐bearing forces. Because cartilage thickness varies over the joint and because the mechanical behavior of cartilage is anisotropic and not uniformly homogeneous over the surface of the joint, biomechanical properties optimally require spatial evaluation. Thus, to determine the impact of compromised cartilage mineralization on cartilage stiffness and ability to respond to compressive stress, topographical structural stiffness mapping of the proximal medial and lateral tibial plateau was assessed by microindentation testing. A constant displacement was applied, and the force needed to maintain the displacement was measured and recorded as peak stiffness. Each mapping locus was acquired at the same perpendicular indentation velocity and amplitude of 30 μm, resulting in an estimated 20%–30% cartilage strain. Peak strain was achieved within 21 seconds of indentation for all samples, consistent with other studies.^(^ ^23^ ^)^ Representative stiffness topographical maps of the tibial plateau of Hyp and WT mice are shown in Figure 2*A*, *B*. Data were binned from a lower‐limit stiffness range of <0.9 N/mm to an upper limit of >6.3 N/mm and individual samples reported as the average tibial structural stiffness over the acquired stiffness range. As shown in Figure 3, compared with WT mice, mean structural stiffness in response to loading of Hyp tibial cartilage was significantly compromised (*p* = 0.0014).

Having confirmed prior findings that mineralization of articular cartilage in Hyp mice is sensitive to replacement therapy with calcitriol and oral Pi,^(^ ^4^ ^)^ we next tested the impact of therapy on the structural stiffness of articular cartilage (Fig. 2*C*). Relative to untreated Hyp mice, there was a significant increase in stiffness in treated Hyp mice (Fig. 3, *p* < 0.0001). In addition, mean stiffness of Hyp‐treated mice was restored to within the range of WT controls (Fig. 3, *p* > 0.05). Upper and lower data ranges for all study groups are summarized in Table 3, including improvement in the upper limit of stiffness in treated Hyp mice.

**Table 3:** Mean Cartilage Stiffness

Sample	Mean stiffness (SD) (N/mm)	Lower limit σ (SD)	Upper limit σ (SD)	*p*
WT (*n* = 6)	2.65 (0.95)	0.3 (0.47)	7.33 (3.86)	
Hyp (*n* = 6)	0.87 (0.33)	0.02 (0.01)	2.11 (1.14)	0.0014^a^
Hyp + Tx (*n* = 12)	2.15 (0.38)	0.03 (0.02)	6.41 (1.84)	<0.0001^b^; >0.05^c^

Abbreviation: WT = wild type.

^a^ WT control versus Hyp‐untreated.

^b^ Hyp‐untreated versus Hyp‐treated.

^c^ Hyp‐treated versus WT.

**Fig. 4:**
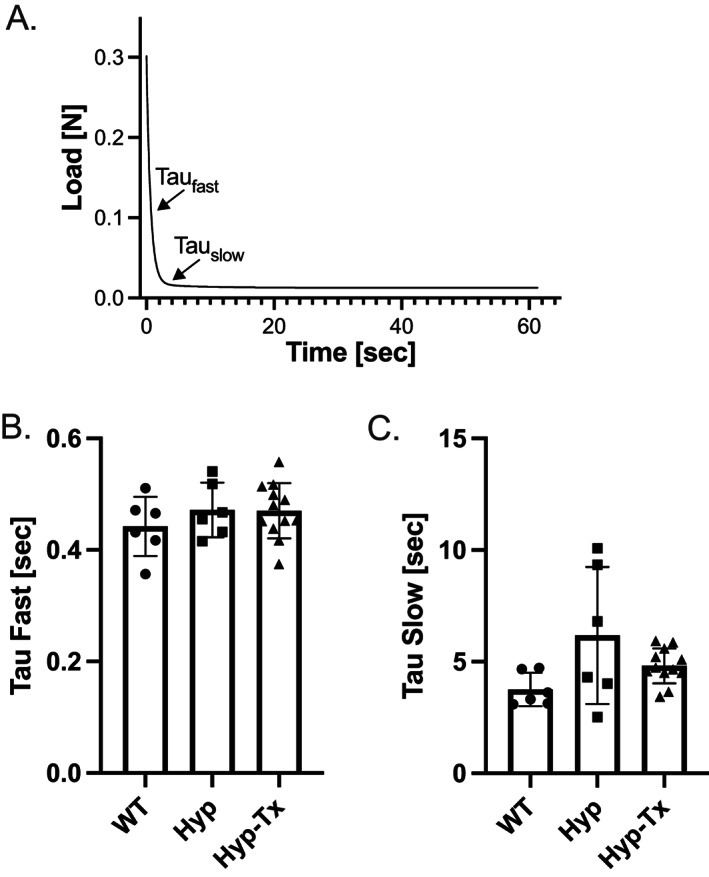
Stress relaxation curves measured from peak load in response to a constant indentation amplitude of 0.03 mm. (*A*) Representative curve illustrating the intrinsic ability of cartilage to relax, or dissipate stress, upon loading analyzed by nonlinear regression and fit with a two‐phase exponential decay formula yielding two time constants, a fast component (Tau_fast_) and a slow component (Tau_slow_). (*B*) Relative to wild type (WT), no significant differences in average Tau_fast_ values were detected in either untreated or treated Hyp mice (*p* > 0.05). (*C*) Compared with WT controls, while average Tau_slow_ values trended toward a delayed rate of relaxation in untreated Hyp mice and sample variance approached WT values in treated Hyp mice, mean values were not significantly different (*p* > 0.05).

**Fig. 5:**
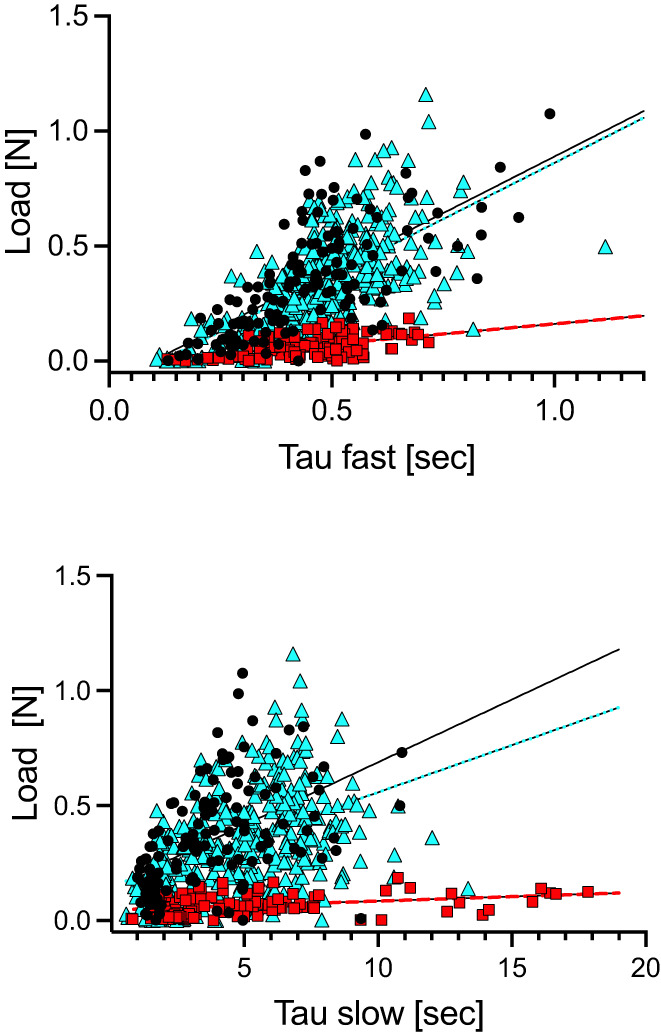
Peak load [N] measured at every microindentation point across the tibial plateau for WT, Hyp, and Hyp‐treated groups, plotted as a function of Tau_fast_ or Tau_slow_ for each respective microindentation point. (*A*) Linear regression analysis of total data points of peak load versus Tau_fast_ revealed that, relative to WT control mice (black circle), the slope of Hyp mice (red square) was significantly different (*p* < 0.0001) from WT, but not significantly different from Hyp mice treated with phosphate and calcitriol (cyan triangle). (*B*) Linear regression analysis of total data points of peak load versus Tau_slow_ revealed that, relative to WT control mice (black circle), the slope of Hyp mice (red square) was significantly different (*p* < 0.0001) from WT, but not significantly different from Hyp mice treated with phosphate and calcitriol (cyan triangle).

The stiffness of a tissue in response to a mechanical stress allows cartilage to accommodate loading forces, but AC can also function to dissipate stress by virtue of its viscoelastic properties. To investigate the potential impact of changes to the biochemical environment of AC, stress relaxation studies were conducted once peak stiffness values were recorded. Indentation was held and time‐dependent stress decay (stress relaxation) was recorded for 30–60 seconds. Stress relaxation curves were recorded, analyzed by nonlinear regression, and fit with a two‐phase exponential decay formula yielding two‐time constants, a fast component (Tau_fast_) and a slow component (Tau_slow_), shown in Figure 4*A*. Relative to WT controls, no significant differences in average Tau_fast_ values were detected in either untreated or treated Hyp mice (Fig. 4*B*, 0.44 ± 0.05, 0.47 ± 0.05, and 0.47 ± 0.05 seconds, respectively). In addition, compared with WT controls, while average Tau_slow_ values trended toward a delayed rate of relaxation in untreated Hyp mice and sample variance approached wild‐type values in treated Hyp mice, mean values were not significantly different (Fig. 4*C*, 3.76 ± 0.75, 6.17 ± 3.07, and 4.82 ± 0.78 seconds, respectively). Pearson coefficient analysis was also conducted for each cartilage indentation point across the entire tibial plateau and peak load values plotted as a function of either Tau_fast_ or Tau_slow_. Data reveal that peak load values of the indentation points across the tibial plateau positively correlated with both Tau_fast_ and Tau_slow_ in WT mice but not in Hyp mice. However, *r* values trended toward those of WT mice in treated Hyp mice (Table 4). Finally, the peak load value for every experimental indentation data point was plotted as a function of the time constants Tau_fast_ (Fig. 5*A*) or Tau_slow_ (Fig. 5*B*) for all experimental indentation points for WT, Hyp, and Hyp‐treated mice, respectively. Linear regression analysis was performed on peak load versus time constant data and revealed significant differences in the slope of fitted Hyp mice data relative to WT mice in both time constants but not to treated Hyp mice (Fig. 5 and Table 5).

**Table 4:** Correlation Coefficient of Peak Load and Time Constants

Sample	Tf *r* value (SD)	Ts *r* value (SD)
WT (*n* = 6)	0.754 (0.103)	0.673 (0.190)
Hyp (*n* = 6)^a^	0.461 (0.257); *p* = 0.027	0.2077 (0.154); *p* = 0.0009
Hyp‐Tx (*n* = 12)^b^	0.716 (0.218); *p* = 0.047	0.563 (0.78); *p* = 0.001

Abbreviations: Tf = Tau_fast_; Ts = Tau_slow_; WT = wild type.

^a^ *p* value WT versus Hyp.

^b^ *p* value Hyp versus Hyp‐treated.

**Table 5:** Linear Regression Analysis of Peak Load Versus Relaxation Time Constants

	WT	Hyp	Hyp‐treated
Tau_fast_			
Slope	0.986	0.181	0.986
Y‐intercept	−0.096	−0.006	−0.115
1/slope	1.01	5.52	1.01
Tau_slow_			
Slope	0.058	0.003	0.034
Y‐intercept	0.132	0.061	0.183
1/slope	17.3	377.8	29.6

Abbreviation: WT = wild type.

## Discussion

Loss of the anatomic structure and biochemical properties of articular cartilage are characteristic of degenerative osteoarthropathy. Degenerative OA is a common finding in the aging population and is prevalent in relatively younger patients with XLH. Joint disease presenting with thinning of the articular surface can impact the upper extremities and the hip and lower limbs at the ankle and knee joints.^(^ ^5^, ^9^, ^10^ ^)^ Vertebral spondylosis, a degenerative osteoarthritis of the spine, has been reported to affect approximately 40% of XLH subjects studied.^(^ ^10^, ^24^, ^25^ ^)^ There is also a strong relationship between the formation of osteophytes, lateral outgrowths of bone at the margins of the synovial joint, and articular cartilage degeneration.^(^ ^26^, ^27^ ^)^ Findings from this study build upon our prior findings to provide insight into the functional impact of OA that arise due to mechanical alteration of AC.^(^ ^4^ ^)^

### Anatomical considerations

The Hyp mouse has provided some important insight into the phenotypic characteristics of XLH. The most prominent anatomical finding was the relative absence of a mineralized zone of AC composed of hypertrophic chondrocytes.^(^ ^4^, ^22^ ^)^ The cartilage of Hyp mice resembles a uniform tissue of unmineralized cartilage that strongly stains for safranin‐O‐positive sulfated proteoglycans. The mineralized zone of AC is believed to play a role in the adhesion of cartilage to the underlying subchondral bone plate at the cement line and to secure the orientation of cross‐linked collagen fibers between the two different tissue types and to resist shear forces at this interface.^(^ ^28^, ^29^ ^)^ The zone of mineralized hypertrophic chondrocytes is also believed to form a graded transitional zone of intermediate stiffness positioned between the unmineralized AC zone and the subchondral bone plate, each with a distinct biochemical profile.^(^ ^13^, ^14^, ^22^, ^30^ ^)^ Likewise, the subchondral bone plate contributes to force dissipation. As a tissue with an elastic modulus higher than mineralized AC, the subchondral bone plate is composed of trabeculae that are inherently able to dissipate stress and withstand significant strain by virtue of the rod and plate organization, anisotropic and biochemical properties.^(^ ^31^, ^32^ ^)^ In addition, trabecular number and spacing are thought to contribute more significantly to biomechanical strength than do trabecular thickness and mineral density.^(^ ^31^, ^33^ ^)^ Bone microCT values of Hyp mice and treated Hyp mice reported in this study closely agree with previously published values^(^ ^34^, ^35^, ^36^ ^)^ and indicate that treatment with calcitriol and phosphate do not significantly improve either trabecular number or spacing of the subchondral bone. Interestingly, despite this, our data reveal that restoration of a mineralized zone of AC with treatment is an important predictor of AC stiffness, underscoring the importance of this zone of tissue to AC function.^(^ ^13^, ^14^ ^)^ These findings lend credibility to the idea that a function of this narrow, mineralized zone of cartilage is in the reduction of stresses imposed on underlying bone.^(^ ^13^, ^14^ ^)^ It is also possible that a compromised mineralized zone contributes to the subchondral sclerosis observed in patients with OA.^(^ ^5^, ^10^ ^)^ Studies conducted by Jia and colleagues strongly support that subchondral sclerosis occurs secondary to degenerative cartilage loss in murine models of OA and is mediated by the mechanical inhibition of sclerostin, further supported by the absence of OA in *Sost*‐knockout mice.^(^ ^37^ ^)^

The mineralized zone of cartilage is also postulated to contribute to the anatomical creation of a confined compartment at the end of long bones.^(^ ^38^ ^)^ A compromised mineralized zone would limit the ability to generate increased internal pressures within articular cartilage and the generation of maximal tissue stiffness in response to load. This is supported by our finding that peak cartilage stiffness, in the absence of a mineralized zone, is significantly lower than controls. The observation that cartilage stiffness in Hyp mice in response to loading is improved with restoration of the mineralized zone of AC is also consistent with the contribution of the mineralized zone to the generation of tissue stiffness. Again, tissue stiffness in response to load is improved despite modest changes to subchondral bone structure.

**Fig. 6:**
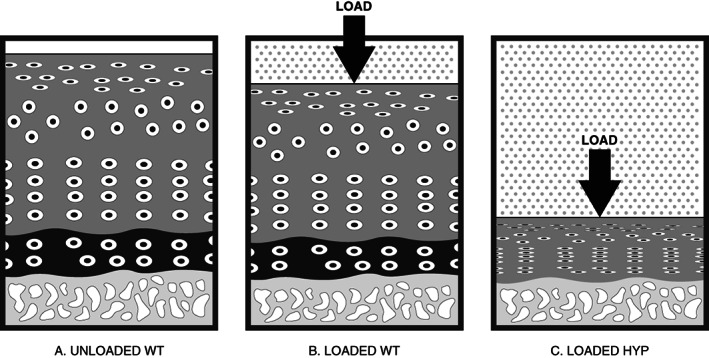
Schematic model showing the response of articular cartilage to equal loading forces. (*A*) WT mouse cartilage showing zones of unmineralized cartilage (dark gray), zone of mineralized cartilage (black) and subchondral bone plate (light gray). (*B*) In WT mice, the mineralized zone imparts resistance to a compressive load, permits the generation of interstitial pressure, and confines the extrusion of water upon compression to the superficial zones of cartilage. (*C*) In Hyp mice, in the absence of a mineralized zone compartment, the tissue is unable to resist an identical compressive load and results in a greater deformation, or stress, of tissue in response to load.

Together, our observation that tissue stiffness is significantly improved compared with untreated Hyp mice while the subchondral bone was not supports the critical role of this narrow zone of mineralized cartilage to the biophysical properties of AC and in buffering the subchondral bone to loading forces. A schematic of these findings is shown in Figure 6.

### Biochemical considerations

The Hyp mouse model previously revealed changes to the ECM that also mirrored alterations to the zonal arrangement of AC.^(^ ^4^ ^)^ The major ECM constituents of articular cartilage are water, collagen, and large proteoglycans, with a lower level of noncollagenous and glycoproteins. In Hyp mice, there is a uniform upregulation of PG biosynthesis throughout the entire cartilage matrix as measured by safranin‐O staining, which quantifiably binds to negatively charged sulfate groups at a 1:1 ratio.^(^ ^4^, ^20^ ^)^ There is also loss of the normal graded PG expression as higher in the unmineralized zone above the tidemark, effectively mimicking an expanded area of unmineralized cartilage.^(^ ^4^ ^)^

The ECM of articular cartilage is negatively charged and hydrated at physiological pH, with compressive strength being determined by the mutual repulsive forces and resistance of polyanionic PGs embedded within a collagen matrix. This tissue composition generates a swelling pressure of resistance to compression.^(^ ^39^, ^40^, ^41^, ^42^ ^)^ Here, we report that the mechanical properties of Hyp mice cartilage are compromised under compression. The loss of a mineralized zone, in the presence of subchondral osteomalacia, limits the generation of increased internal pressures and stiffness in Hyp mice in response to load, consistent with our finding that peak cartilage stiffness is significantly lower than controls. This also translates into a much higher degree of tissue deformation across the tibial plateau in response to loading compared with the stiffer cartilage of control mice or treated Hyp mice. Although the cartilage stiffness of untreated Hyp mice is significantly lower than controls, it would be presumably catastrophic if not for the compensatory upregulation of PGs to withstand compressive forces. The aggregated PG density and the repulsive forces they generate within the collagen matrix would help to resist applied loads.^(^ ^40^ ^)^ However, because Hyp mice lose 50% of cartilage volume by 7 to 8 months of age,^(^ ^4^ ^)^ a finding consistent with the loss of cartilage in adults with XLH,^(^ ^5^, ^6^ ^)^ this compensatory mechanism of sustained elevated levels of PGs is unable to maintain the integrity of the tissue. These findings also underscore the importance of treatment throughout adulthood to maintain the functional role of the articular chondrocyte as maintaining the ECM and structure of a terminally differentiated tissue. Treatment improves mineralization of the deepest zone of cartilage and also stiffness in response to loading, while restoring both the levels and the zonal arrangement of matrix PGs similar to controls.^(^ ^4^ ^)^

Our studies also provide insight into the role of the solid matrix in stress dissipation under compression. Stress relaxation of epiphyseal cartilage occurs in a restricted compartment that is formed by a mineralized zone of cartilage. Compaction of the cartilage matrix decreases the porosity of the solid matrix and increases the frictional resistance to interstitial water flow. The porosity of the solid matrix, in turn, impacts the rate of stress dissipation, which is influenced by PG content. Proteoglycan depletion in cartilage increases porosity and results in decreased peak load and time to equilibrium (ie, faster relaxation times).^(^ ^43^, ^44^ ^)^ Conversely, higher PG concentrations would be predicted to decrease pore size and slow stress relaxation. However, the rates of stress relaxation between untreated Hyp mice, with elevated levels of PGs, and WT mice were not significantly different, although the Tau_slow_ phase of relaxation trended toward slower rates in untreated Hyp mice. Intrinsic differences in the viscoelastic properties between samples are supported by Pearson coefficient analysis of peak stiffness values plotted as a function of either Tau_fast_ or Tau_slow_. There is a strong correlation between peak stiffness and the rate at which the tissue relaxes in response to load in WT mice. Hyp mice, as seen in Figures 3 and 4, never reach stiffness values achieved in WT mice and the correlation between stiffness and the recovery from tissue indentation during the slow phase of relaxation is absent. These data suggest that not only does the mineralized zone allow the tissue to stiffen in response to load but also to recover during the relaxation phase and that loss of the functionality of articular cartilage contributes to the early onset of cartilage degeneration in XLH.

Based on these data, our current model is that the upregulation of PGs and the resulting restriction of fluid flow helps to minimize cartilage deformation in the absence of a mineralized zone and aids in the preservation of stress relaxation. Again, we propose that in the absence of the compensation of higher PG levels in Hyp mice, the rates of cartilage degeneration in XLH would be accelerated. Without the intrinsic property of stress relaxation in a tissue that functions to accommodate a lifetime of imposed forces, cartilage would be expected to degenerate at much higher rates. Future studies aimed at comparing tissue porosity and experimentally reducing PG content in untreated and treated Hyp models will allow further characterization of the cellular and genetic changes that transiently preserve cartilage function in the phosphate‐wasting environment and contribute to the restoration of the hierarchical structure with treatment.

### Limitations

The role of collagen as an important constituent of the cartilage matrix in these studies is currently unknown. The perpendicular arrangement of collagen as AC interfaces with the subchondral bone is believed to be supported by the mineralized zone of AC. Although there is no evidence that type II collagen expression is affected in Hyp mice or patients with XLH, the loss of a mineralized zone of AC may also introduce additional mechanical deficiencies to collagen in the ability of AC to withstand interface stress. Type X collagen is highly expressed by terminally differentiated AC chondrocytes and plays a role in physiological mineralization and is likely to play a significant role once the zonal arrangement of mineralization is restored, where mineralization will substantially increase matrix stiffness.^(^ ^45^, ^46^ ^)^

### Conclusions

The Hyp murine model allowed a unique examination of several interesting physiological questions, including an opportunity to better understand the sequelae of subchondral sclerosis in the setting of OA, a coincident finding in both the XLH and the general aging population.^(^ ^2^, ^5^, ^9^, ^10^, ^47^, ^48^ ^)^ Our data suggest that while the upregulation of PG biosynthesis allows the viscoelastic properties of cartilage to persist, it is the absence of the mineralized zone that significantly contributes to the inability of the tissue to stiffen in response to load. In addition, the Hyp mouse model may lend to a better understanding of the contributions of the mineralized zone and PGs to viscoelasticity, a property that subserves the function of articular cartilage at the ends of long bones.

Next, our findings help to better illuminate the etiology of OA.^(^ ^49^ ^)^ Articular chondrocytes are tasked with lifelong production of extracellular matrix that has evolved to adapt to loading forces imposed on the synovial joint. The treatment of AC degeneration in any patient population is a significant clinical challenge because of the lack of the intrinsic regenerative capacity of cartilage, an avascular tissue populated by terminally differentiated chondrocytes. Therapies have focused on symptom and pain management, until the necessity of joint replacement surgery is reached. Surgical interventions are not as widely afforded to patients with XLH, who progress to earlier end‐stage OA, although they may benefit from knee or hip arthroplasty.^(^ ^50^ ^)^ The early‐onset presentation of OA in adults with XLH, relative to the unaffected aging population,^(^ ^5^ ^)^ suggests an etiology unique to XLH. Indeed, it differs significantly from that of OA in the general population that is characterized by cartilage thinning, PG loss, and damage to the collagen network. The relative improvement of structural stiffness and the mineralized zone of AC with therapy strongly support a primary role of the phosphate‐wasting environment in the etiology of OA. Our findings also challenge the belief that abnormal alignment and distribution of load on weight‐bearing joints as the sole cause of OA in these patients. Importantly, these data suggest that early and sustained treatment to maintain the biosynthetic capacity of articular chondrocytes throughout life is an important consideration to delay or prevent OA and associated osteophytes.^(^ ^26^ ^)^ Data from phase 3 burosumab studies in adults with XLH show improvement in healing osteomalacia and pseudofractures and contributed to improved ambulatory function.^(^ ^51^ ^)^ Although it remains to be seen if this therapy introduced early in life slows the progression of OA, our data suggest that the combination of improved mineralization of tissues, along with increased engagement of weight‐bearing activities, may lessen the physical burden of XLH in adulthood. Future studies will also focus on therapies that can improve mineralization of both AC and the subchondral bone plate to determine if the function and mechanical properties of AC can be restored to near normal.

## Author Contributions

**Carolyn M Macica:** Conceptualization; formal analysis; funding acquisition; investigation; methodology; project administration; resources; validation; writing – original draft; writing – review and editing. **Steven M Tommasini:** Formal analysis; investigation; methodology; writing – review and editing.

## Disclosures

All authors state that they have no conflicts of interest.

### Peer Review

The peer review history for this article is available at https://www.webofscience.com/api/gateway/wos/peer-review/10.1002/jbm4.10796.

## Data Availability

Some datasets generated during and/or analyzed during the current study are not publicly available but are available from the corresponding author on reasonable request.
